# Obesity-Related Behaviors among Poor Adolescents and Young Adults: Is Social Position Associated with Risk Behaviors?

**DOI:** 10.3389/fpubh.2015.00224

**Published:** 2015-10-16

**Authors:** Miranda Lucia Ritterman Weintraub, Lia C. Fernald, Elizabeth Goodman, Sylvia Guendelman, Nancy E. Adler

**Affiliations:** ^1^Public Health, Touro University California, Vallejo, CA, USA; ^2^Public Health, University of California Berkeley, Berkeley, CA, USA; ^3^Goodman Lab, General Academic Pediatrics, Center for Child and Adolescent Health Research and Policy, Mass General Hospital for Children, Boston, MA, USA; ^4^Department of Psychiatry, University of San Francisco, San Francisco, CA, USA

**Keywords:** Mexico, socioeconomic status, social position, relative deprivation, obesity, obesity-related behaviors, poverty

## Abstract

**Highlights**
- Different measures of social position capture unique dimensions of relative rank among youth.
- Youth-specific measures of social position may be important in identifying the most at-risk for obesity.
- Lower social status youth are more likely to be at-risk for obesity-related behaviors compared to those with a higher rank.

This cross-sectional study examines multiple dimensions of social position in relation to obesity-related behaviors in an adolescent and young adult population. In addition to using conventional measures of social position, including parental education and household expenditures, we explore the usefulness of three youth-specific measures of social position – community and society subjective social status and school dropout status. Data are taken from a 2004 house-to-house survey of urban households within the bottom 20th percentile of income distribution within seven states in Mexico. A total of 5,321 Mexican adolescents, aged 12–22 years, provided information on obesity-related behaviors (e.g., diet, physical activity, sedentary behavior) and indicators of subjective and objective social position. A parent in each household provided information on socioeconomic status of the parent and household. Ordinal logistic regressions are used to estimate the associations of parental, household and adolescent indicators of social position and obesity-related risk behaviors. Those adolescents with the highest odds of adopting obesity risk behaviors were the ones who perceived themselves as lower in social status in reference to their peer community and those who had dropped out of school. We found no significant associations between parental education or household expenditures and obesity-related risk behaviors. Immediate social factors in adolescents’ lives may have a strong influence on their health-related behaviors. This study provides evidence for the usefulness of two particular measures, both of which are youth-specific. Adolescents and young adults who have dropped out of school and those with lower perceived relative social position within their community are more likely to be at-risk for obesity-related behaviors than those with higher relative social position. We conclude that youth-specific measures may be important in identifying the most at-risk among relatively homogeneous populations of youth.

## Introduction

Obesity prevalence is growing among teens in Mexico. In 2000, both the Centers of Disease Control and Prevention and the International Obesity Task Force classified approximately 19% of Mexican adolescents aged 10–17 as overweight or obese (1). By 2006, the National Survey for Health and Nutrition, found that up to 23% of Mexican adolescents aged 12–19 were overweight or obese (2). Obesity and overweight during adolescence are highly predictive of serious health risks throughout adulthood, including adult obesity and diseases, such as diabetes, hypertension, and cardiovascular disease (3, 4).

Underlying the trends in weight gain within developing countries, such as Mexico, undergoing rapid nutrition transitions, are sharp increases in availability and consumption of high fat and refined carbohydrate products and sugars, snack foods, and high calorie sodas, declines in physical activity, and increases in time spent on sedentary behaviors associated with mass media technology (5–16).

These shifts in obesity are socioeconomically patterned (15, 17, 18). Fernald (19) found a positive association between socioeconomic status and obesity within low-income adult populations in Mexico, although the reverse was true among children (20). Within Brazil, an upper-middle-income country like Mexico, a positive association between overweight and household assets was found among a low-income indigenous population (21). These studies have examined the links between obesity and social position among Mexican children and Mexican adults, but no data have been published on associations between social position and obesity risk among Mexican adolescents. Adolescence is the transitional period from childhood to adulthood associated with rapid change in behaviors. Adult habits may get initiated in adolescence, but social hierarchies that influence adolescent behavior may differ from those that influence adult behavior. Adolescent and young adult social status may not only be influenced by the SES of the family of origin, but by their peer networks as well (22). Variations in social position among Mexico’s poor adolescents and young adults help identify who is most at risk among this already high-risk group and inform efforts at prevention.

**Figure 1:**
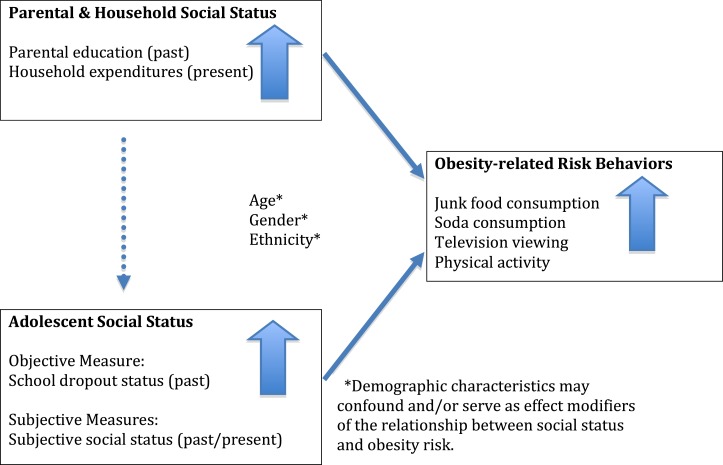
**A psychosocial model for obesity risk among the poor during adolescence**.

Different measures of social position – parental education, household expenditures, community and society subjective social status, and school dropout status – each capture unique aspects of the multi-dimensional nature of adolescent and young adult social position (23). Parental education may reflect levels of knowledge that affect parenting practices, while household expenditures capture material resources available in the adolescent’s household. Subjective social status involves the cognitive averaging of multiple factors that may be associated with either parental status and the adolescent’s own, while school dropout is a characteristic of the adolescent that shapes current conditions. Parental, household, and youth-specific measures of social position may all be associated with obesity risk, as illustrated in Figure 1.

Youth-specific measures of social position and educational status appear to be more strongly associated with risk behaviors than are parental and household measures. Among public school students in Morelos, Mexico, aged 11–19, a composite SES indicator, based on household belongings and parental income, was not correlated with body mass index, while, controlling for age, students with more years of education were less likely to be overweight (24). In a US study of upper-middle class junior and senior high school students, Goodman et al. (25) found no association of obesity with parental education or household income, controlling for age, school site, number of people in the house, and race-by-gender group, but found a significant inverse relationship between obesity and students’ perceived community standing within their school (25). These findings suggest that youth-specific measures may be better equipped to tap into the social hierarchies most relevant to adolescents, and be more predictive of future health outcomes than are parental measures.

By identifying links between social position and obesity-related behaviors among the poor in Mexico, we aim to provide insight into the pathways between relative deprivation and health-related behaviors among adolescents. We also aim to examine whether perceived social status may detect nuanced variations in social position that are not captured when using standard SES measures. We studied a sample of urban adolescents and young adults from households defined as being in the bottom 20th percentile of the income distribution in Mexico and examined multiple dimensions of social position in relation to obesity-related behaviors. A relatively homogeneous population of poor people allows for the study of more nuanced social position variations.

We identify the adolescents and young adults at highest risk of reporting behaviors that have been independently linked with obesity in Mexico and the US. These include the consumption of sweetened, carbonated beverages and high calorie, low nutrient food, television viewing, and lack of physical activity (10, 15, 26–29). We hypothesize that the association between social position and health-related behaviors in this low-income sample will be positive (see Figure 1), matching the pattern seen among the low-income adult population of Mexico (19, 30). The identification of social patterns associated with obesity-related behaviors among high-risk adolescents and young adults within countries undergoing nutritional transitions may help inform interventions to prevent obesity and its associated co-morbidities during adulthood.

## Materials and Methods

### Procedure (study design and sampling)

This cross-sectional study used data from the 2004 evaluation of the Mexican government’s poverty alleviation program, Oportunidades. In the 2004 program, urban areas (defined as having 50,000–1 million inhabitants) within seven states in Mexico with the highest density of eligible households (≥500) were selected and matched to comparison areas for evaluation purposes. Following this process, a random set of census tracts was identified within the areas with probability proportional to size. From this sample of 204 urban areas, a random sub-set of 157 areas was selected for the adolescent risk behavior component. Each household was visited up to three times to identify adolescents and young adults, aged 12–22, and to collect household SES and adolescent risk behavior data.

Households enrolled in Oportunidades who had adolescents enrolled in school up to the 12th grade received bi-monthly cash transfers. These education grants served to support youth in completing high school. Regular school attendance was not enforced among youth in the Oportunidades program, however, it was a requirement for continued educational grant support (31). In addition to the education grants, a point system was introduced in 2003 whereby for every grade completed, from 9th to 12th grade, a certain number of points were issued to the student. Once the student completed high school, as long as they did so before the age of 22, they could transfer their points into pesos after 2 years or they could use the points immediately to attend college, purchase health insurance, receive a business loan or get credit for housing (31). Although students in grades 10–12 were required to attend eight health education sessions each school year (31), no weight and height data were collected for this study sample.

A total of 7,900 adolescents were identified at baseline. Of this group, 5,321 adolescents (67%) had complete data on adolescent subjective social status, school dropout status, demographic characteristics, obesity-related behaviors, and parental, household, and neighborhood data. Compared to adolescents who were included in the study, those that were excluded due to incomplete data were more likely to be female (*p* = 0.002), older (*p* < 0.0001), indigenous (*p* = 0.018), school dropouts (*p* < 0.0001), have less educated parents (*p* < 0.0001), be from households with lower monthly expenditures per person (*p* < 0.0001), and more likely to have lower community social status scores (*p* = 0.0001).

Approval for the 2004 study was obtained by the Research Committee at the National Institute of Public Health in Mexico, and by the Committee on the Protection of Human Subjects at the University of California at Berkeley. A detailed explanation of the survey procedures was given and an informed consent declaration was obtained prior to participation. Both parental consent and youth assent were obtained for adolescents under 16 years of age. Data for the adolescent survey were collected using an audio-computer assisted self-interview system. A supplemental survey on household and parental socioeconomic status was also administered to the parents.

Adolescent survey data included information on obesity-related behaviors, social position (community and society subjective social status, and school dropout status), and demographic characteristics.

### Measures

#### Adolescent Socioeconomic Position (Adolescent Self-Report)

##### Perceived social position indicators

A modified version of the Subjective Social Status (SSS) Scale-Youth Version was completed (32). Two 10-rung ladders were depicted, one of which was the standard Society SSS scale, while the other was new for this 2004 evaluation study. For the ladder scales, the top represents those with the highest ranking and the bottom represents those with the lowest ranking. The standard *Society SSS* question asked adolescents to make a relative socioeconomic comparison of their current household with “all households in Mexico.” A second question, *Community SSS*, asked adolescents to rank themselves compared to their close group of friends according to their level of relative importance. Specifically, they are asked, “On which rung of the ladder are you in comparison to your group of friends?” At the top of the ladder are the “most important of the group” and at the bottom of the ladder are the “least important of the group.”

##### Objective social position indicator

Adolescents were also asked whether they had dropped out of school (yes/no).

#### Sociodemographic Covariates (Adolescent Self-Report)

Adolescents provided data on age (continuous), gender (male/female), and indigenous status (“yes” defined as speaking an indigenous language in addition to Spanish).

#### Objective Parental and Household Socioeconomic Position (from Parents’ Survey)

##### Maternal and paternal education

Maternal education and paternal education were each first separately categorized into dichotomous variables: high school education or above (yes/no). Subsequently these categories for each parent were pooled to create a new three-category indicator of parental high school education: neither parent, one parent, or both parents.

##### Total monthly household expenditure

Total monthly household expenditures were estimated by adding parents’ reports of “household reported weekly expenditure on food items,” “monthly expenditure on non-food items,” including services, goods, such as tobacco, and rent, and was divided into quartiles (0–25%, 26–50%, 51–75%, 76–100%).

#### Adolescent Obesity-Related Behaviors (Adolescent Self-Report)

Adolescents were asked how many bags of chips, packets of cakes or sweet breads, and packets of sweets they consumed and how many sodas during the prior day. The authors made two binary variables: junk food, defined as food of minimal nutritional value (“yes” defined as one or more portions of cake, sweets or chips) and soda consumption (“yes” defined as one or more carbonated, sweetened beverages). Adolescents were also asked the number of hours they watched television during their last viewing. Adolescents who reported viewing television 3 h or more were classified as television watchers. Adolescents were also asked the number of days they exercised during the previous week. A dichotomous variable was created, categorizing adolescents into those who reported ever having exercised during the previous week and those who did not. These four obesity-related behaviors were then combined to create an obesity risk index based on the adoption of 0–2, 3, or 4 of these behaviors. This index was designed to combine dietary and activity patterns associated with the nutritional and economic shifts of middle-income countries (15) to predict obesity risk among Mexican adolescents.

### Statistical analysis

Descriptive statistics were generated of the study sample, and we calculated the proportion of adolescents within each obesity-related behavior as categorized by the obesity risk index.

To examine the associations between the obesity risk index and gender, indigenous status, school dropout status, parental high school education, and household expenditures, we conducted Chi-square analyses. The difference in the proportion of adolescents within each above-mentioned covariate category was calculated according to whether they were classified as having adopted 0–2, 3, or 4 obesity-related risk behaviors. Analysis of variance (ANOVA) was used to determine the difference in mean age, society SSS, and community SSS according to each of the three obesity risk index categories. Spearman correlations were run to examine the correlations between each of the social position indicators, with the exception of school dropout status. The bivariate associations between school dropout status and the other social position indicators were examined using Kruskal–Wallis equality-of-populations rank tests for continuous indicators and Chi-square analyses for categorical indicators.

To examine the association between the 3-category obesity risk index (0–2, 3, or 4 behaviors) (dependent variable), and social position (independent variables), we used ordinal logistic regression analyses. Two tests were conducted to ensure that the proportional odds assumption was not violated. To explore differences in association between parental and adolescent social position indicators, parental and adolescent indicators were examined both separately and together. The first model examines the independent association between parental and household social position indicators and the obesity risk index. The second model examines the association between school dropout status, an objective adolescent measure, and obesity risk. The third model examines the association between adolescent SSS and obesity risk. The final model includes all social position indicators. All analyses controlled for age, gender, indigenous status, welfare status (adolescents from recipient households of the Mexican government’s poverty alleviation program, “Oportunidades”), the fixed effect of state (adolescent residency in one of seven sample states in Mexico), and clustering at the neighborhood level.

Statistical analyses were conducted using STATA 10.1 (STATA Corporation, College Station, TX, USA).

## Results

### Description of study variables

The mean age of the sample is 17.11 ± 2.04 years and has slightly more females (52.18%) than males (Table 1). Only 4.2% of the sample is indigenous and 44.7% of adolescents are school dropouts. For 74.7% of the sample, neither parent has received a high school education, compared to 6.1%, for whom both parents had a high school education. The mean ranks for society and community SSS are 5.36 (SD = 2.35) and 5.04 (SD = 2.36), respectively.

**Table 1:** **Demographic and social position measures of adolescent study participants (*n* **=** 5,321)**.

Covariates	Total (%)	Mean (SD)^b^
**Demographic characteristics**		
Age (12–22 years)		17.11 (2.04)
Gender		
Male	2,540 (47.8)	
Female	2,772 (52.2)	
Indigenous		
No	5,088 (95.8)	
Yes	224 (4.2)	
**Adolescent social position indicators**		
Adolescent dropout status		
No	2,936 (55.3)	
Yes	2,376 (44.7)	
Society subjective social status^a^ (1–10)		5.36 (2.35)
Community subjective social status^a^ (1–10)		5.04 (2.36)
**Parental and household social position indicators**		
Parents high school education		
Neither	3,970 (74.7)	
One	1,016 (19.1)	
Both	326 (6.1)	
Household expenditures		
0–25	1,323 (24.9)	
26–50	1,299 (24.5)	
51–75	1,364 (25.7)	
76–100	1,326 (25.0)	

*^a^Both society SSS and community SSS are represented by a 10-rung ladder. The society SSS ladder asks adolescents to locate their family in relation to other families in Mexican society. The community SSS ladder asks adolescents to rank themselves in comparison to their group of friends. A 10 represents those with the highest ranking and a 1, those with the lowest ranking*.

*^b^Standard deviation (SD)*.

Over half of the adolescents in our sample (55.6%) reported 0–2 obesity-related behaviors (2.6, 14.6, and 38.5% reported 0, 1, and 2 behaviors, respectively). 28.2% reported three behaviors and 16.1% reported engaging in all four obesity-related risk behaviors. All of those who reported adopting three or more behaviors reported soda consumption (Table 2).

**Table 2:** **Proportion (%) of obesity-related risk behaviors within each category of the obesity risk index (*n* **=** 5,321)**.

Obesity risk index	Total (%)	Four obesity-related behaviors							

		Junk food		Sodas		TV		Exercise	

		None	**≥**1	None	**≥**1	**<**3 h	**≥**3 h	No	Yes
0–2 behaviors	2,961 (55.6)	55.4	44.6	58.1	41.9	59.0	41.0	47.3	52.7
3 behaviors	1,498 (28.2)	31.4	68.6	0.0	100.0	30.6	69.4	62.1	37.9
4 behaviors	853 (16.0)	0.00	100.0	0.0	100.0	0.0	100.0	100.0	0.0

*Binary variables were made to categorize aspects of adolescents’ diet, sedentary behavior, and physical activity level. Junk food was based on whether chips, cakes, sweet breads, and sweets were consumed yesterday. Soda was based on whether soda was consumed yesterday. Television viewing was used to capture sedentary behavior and was categorized by the number of hours the adolescent reported watching during their last viewing. Exercise was defined as adolescent report of physical activity on any day during the previous week*.

More obesity-related risk behaviors were reported by females compared with males (*p* < 0.0001) and by non-indigenous versus indigenous adolescents (*p* < 0.0001) (Table 3). Age was not associated with obesity risk. More obesity-related behaviors were reported in adolescents who had dropped out of school compared with those who had not (*p* < 0.0001). Obesity risk behaviors were not significantly related to adolescents’ parents’ high school education, household expenditures, or society SSS. However, a greater number of obesity risk behaviors were associated with a lower mean community SSS score (*p*-value < 0.0001).

**Table 3:** **Chi-square and analysis of variance tests show bivariate associations between covariates and obesity-related behaviors (*n* **=** 5,321)**.

Covariates	Obesity risk index^a^ (0–2 vs. 3 vs. 4 risk behaviors)			*p*-value^c^

	0–2	3	4	

	*n* (%)^b^	*n* (%)	*n* (%)
Total	2,961 (55.6)	1,498 (28.2)	853 (16.0)	
Gender				<0.0001
Male	1,410 (47.6)	774 (51.7)	356 (41.7)	
Female	1,551 (52.4)	724 (48.3)	497 (58.3)	
Indigenous				<0.0001
No	2,808 (94.8)	1,456 (97.2)	824 (96.6)	
Yes	153 (5.2)	42 (2.8)	29 (3.4)	
Adolescent dropout status^e^				<0.0001
No	1,745 (58.9)	811 (54.1)	380 (45.6)	
Yes	1,216 (41.1)	687 (45.9)	473 (55.5)	
Parents high school education				0.379
Neither	2,188 (73.9)	1,123 (75.0)	659 (77.3)	
One	586 (19.8)	286 (19.1)	144 (16.9)	
Both	187 (6.3)	89 (5.9)	50 (5.9)	
Household expenditures				0.368
0–25	739 (25.0)	354 (23.6)	230 (27.0)	
26–50	732 (24.7)	263 (24.2)	204 (23.9)	
51–75	776 (26.2)	379 (25.3)	209 (24.5)	
76–100	714 (24.1)	402 (26.8)	210 (24.6)

	**Mean (SD)^b^**	**Mean (SD)**	**Mean (SD)**	***p*-value^d^**

Age (12–22 years)	17.10 (2.04)	17.11 (2.03)	17.18 (2.02)	0.5519
Society subjective social status (1–10)	5.40 (2.34)	5.32 (2.31)	5.28 (2.42)	0.3137
Community subjective social status (1–10)	5.16 (2.40)	4.97 (2.28)	4.75 (2.33)	<0.0001

*^a^The obesity risk index is based on the number of obesity risk behaviors (0–2, 3, or 4) adopted by the given adolescent. The behaviors used to create the index are: consumption of one or more sodas yesterday; consumption of one or more servings of junk food yesterday, watching three or more hours of television during the last viewing, and not exercising at all during the past week*.

*^b^Sample size (n); proportion (%); SD*.

*^c^Chi-square tests were used to determine the proportion of adolescents within each obesity risk category according to the categories of each categorical variable*.

*^d^Analysis of variance (ANOVA) was used to determine whether continuous covariates differed across categories of obesity risk. All values have two degrees of freedom*.

*^e^Adolescent dropout status is an objective measure of adolescent social position*.

### Correlates of social position indicators

According to Bonferroni-adjusted Spearman correlations (results not shown), all presented indicators of social position, with the exception of society SSS, had small to moderate significant correlations (*p* < 0.05) with each other. Society SSS was significantly correlated only with community SSS (*r* = 0.25). According to Kruskal–Wallis equality-of-populations rank tests in our sample (results not shown), school dropout status is significantly associated with community SSS (*p* = 0.0001) and is also significantly associated with parental high school attainment (*p* < 0.0001) and total monthly household expenditures (*p* < 0.0001) (Chi-square test results not shown).

### Associations between social position and obesity risk index

The obesity risk index was positively associated with having dropped out of school (OR = 1.43; 95% CI: 1.27–1.62) and negatively associated with having a higher community SSS rank (OR = 0.96; 95% CI: 0.94–0.99) (Table 4). No other indicators showed a significant association with obesity risk behaviors, including parental education or household expenditures. Only the final model is presented, as the results are consistent with the first thee models. All models controlled for age, gender, indigenous status, welfare status, fixed effect of state, and clustering at the community level. There was no effect modification of the social position-obesity risk associations by age, gender, and ethnicity in any of the regression analyses. In supplementary logistic regression analyses (data not shown) being a school dropout was significantly associated with a lower odds of being physically active (OR = 0.43; 95% CI: 0.38–0.49) and an increased odds of watching three or more hours of television during last viewing (OR = 1.24; 95% CI: 1.10–1.40). Higher community SSS was significantly associated with increased odds of being physically active (OR = 1.06; 95% CI: 1.04–1.09). School dropout and community SSS were not significantly associated with the diet behaviors.

**Table 4:** **Adjusted^a^ odds ratios using ordinal logistic regression analyses of the associations between multiple indicators of social position and the obesity risk index with three behavior categories: 0–2, 3, or 4^b^ (*n* **=** 5,321) (odds ratios and 95% confidence intervals)**.

Social position variables	All indicators
**Parental and household social position**	
Household expenditures (reference = 0–25%)	
26–50%	0.94 (0.81–1.10)
51–75%	0.94 (0.80–1.11)
76–100%	1.02 (0.88–1.20)
Parental high school (reference = neither)	
One parent	0.97 (0.83–1.15)
Both parents	1.01 (0.80–1.26)
**Objective adolescent social position**	
School dropout status (reference = no)	1.43** (1.27–1.62)
**Subjective adolescent social position**	
Society subjective social status (continuous)	0.99 (0.97–1.01)
Community subjective social status (continuous)	0.96** (0.94–0.99)

**p < 0.01.

*p < 0.05.

*^a^All models controlled for age, gender, indigenous status, fixed effect of state, Oportunidades-recipient status, and clustering at the neighborhood level*.

*^b^The outcomes for the three ordinal logistic regression models are the obesity risk index based on number of obesity risk behaviors (0–2, 3, or 4)*.

## Discussion

Our findings show that, within the poorest quintile of the urban Mexican adolescent and young adult population, both objective and subjective measures of social position were associated with diet and physical activity. Contrary to our hypotheses, increased risk of obesity-related behaviors was associated with lower social position. Specifically, school dropout status, a measure of objective social position, and perceived lower status within the adolescent’s community (community SSS), a measure of subjective social position, were independently associated with an elevated risk of obesity-related behaviors, with school dropout status having the strongest association.

Our findings of low SES youth show consistencies with other cross-sectional studies of adolescents in Mexico and the United States from the full range of socioeconomic groups. These also show youth-specific measures to be more strongly associated with obesity risk behaviors compared to parental and household measures (24, 25). Although we found that adolescents from households whose monthly expenditures were in the top quintile within this generally impoverished group were significantly more likely to consume soda (OR = 1.41; 95% CI: 1.18–1.68) in supplementary analyses, monthly household expenditures were not significantly associated with the obesity risk index. In contrast, staying in school and perceiving oneself as ranked high within the community were associated with decreased risk of adopting obesity-related behaviors.

Notably, only youth-specific measures of adolescent social position maintained an independent association to obesity risk behaviors in our study. Unlike a previous study (20) that explored the association between risk of obesity and maternal social position among preschoolers living in Mexico, parental education and household expenditures did not uniquely contribute to obesity risk behaviors among adolescents in the current analysis. This is consistent with an understanding of adolescence as a period of transition from parental to peer influence and suggests that adolescents may show different patterns of influence from different aspects of social hierarchies than either younger children or adults. That this study found obesity risk behaviors associated with adolescent measures of social position as opposed to household and parental measures of social position provides additional evidence for the importance of the more proximate and specific influence of peer networks on adolescent social position (25). Furthermore, it may suggest the presence of misclassification bias when using parental and household measures of SES to capture adolescent social position.

Findings related to peer networks and adolescent risk behaviors might provide a good model for how to think about the association between youth-based measures of social position and obesity risk behaviors. Unlike parental and household social position measures, youth-specific measures, such as community SSS and school-dropout status, tap into adolescents’ peer networks. Among a nationally representative sample of US adolescence, youth who were socially isolated and had fewer friends were more likely to be overweight compared with those who were socially integrated (33). Socially isolated adolescents are more likely to view more hours of television and less likely to participate in sport-related activities (33). The same social processes may be occurring among Mexican adolescents. In supplemental analyses, we found that the youth-specific community SSS and school dropout measures were more strongly associated with hours of television viewing and physical activity than with junk food and soda consumption. School dropout status was more strongly associated with obesity risk behaviors than was the community SSS ladder. This may be explained by the social price youth pay for discontinuing their education, including the breakdown of peer networks. Further research into the social environment in which obesity-related behaviors are adopted among poor Mexican adolescents would be needed to address this question.

Finally, our findings also demonstrate that social stratification, as measured by perceived social status, may be able to detect nuanced variations in social position that may go undetected when using standard measures of social position among relatively homogenous socioeconomic cohorts. This is consistent with another study using the same Mexican adolescent cohort, which found a more consistent association between measures of perceived social status and substance use than standard objective measures (22). Our study provides evidence of the utility of subjective measures of social position when studying socioeconomic variations in a group with little socioeconomic variability. Further, the community SSS measure may have reflected a hierarchy with greater variability than that of society SSS. This may explain the non-significant association between the society SSS ladder and the obesity risk index.

Some clear limitations are evident in this study. First, there was no data on the weight status of the adolescents and extensive questions regarding dietary intake, physical activity, and sedentary behavior were not included in the house-to-house survey. Misclassification may have occurred, as we relied on self-reported measures and dietary recall was based on the previous day, as opposed to the previous week. We used consumption of food of minimal nutritional value and carbonated, sweetened beverages as a proxy for dietary intake, exercise during the past week as a proxy for physical activity, and time spent watching television as a proxy for sedentary behavior. These measures are not as sensitive as measures of caloric intake, fitness and more detailed measures of sedentary behavior (34–36). Further, there was no information on whether or not each household had a television, although it is estimated that approximately 90% of urban households living in poverty in 2004 owned a television (37). Second, due to the adolescents dropped from the study, the effect of social position on obesity risk behaviors might be conservative, underestimating the association between low social position and increased risk of obesity-related behaviors. Third, we did not have data on food insecurity and hunger. When we added total monthly food expenditures per person into our regression models, effect sizes of associations did not change. Fourth, the findings are limited in generalizability to Mexican adolescents living in the bottom twentieth percentile of wealth in urban areas in Mexico. Lastly, due to the cross-sectional nature of our study, we cannot determine the direction of the associations. For example, it is possible that adolescents who have dropped out of school and who rank themselves low on the community SSS ladder are more likely to be overweight, or have a higher obesity risk. Reverse causality would provide an additional explanation for why we did not find evidence of the effect of parental SES and household expenditures on the obesity risk index.

The prevalence of adolescent obesity-related behaviors is increasing worldwide, including significant rises in low-income and middle-income countries (38, 39). The short- and long-term health consequences associated with these behaviors, which are close determinants of adolescent obesity, not only reduce the quality of life and life expectancy, but also place a burden on health services (38). Understanding the risk factors of obesity in the context of poor urban adolescent populations is a critical first step in developing effective interventions to reverse these trends.

In conclusion, this study suggests an inverse social gradient in adolescent and young adult obesity-related behaviors among a socioeconomically homogenous cohort, based on both an objective and a subjective youth-specific measure of social position. It provides additional evidence that different measures of social position capture unique dimensions of relative rank among youth, and vary in their ability to detect nuanced variations in social position among a relatively homogeneous socioeconomic cohort. Measures of adult social position may be inappropriate for capturing adolescent social position. More research is needed to establish youth-specific measures of social position, and to understand the links between social position and health-related risk behaviors among adolescents in diverse national and socioeconomic contexts.

## Conflict of Interest Statement

The authors declare that the research was conducted in the absence of any commercial or financial relationships that could be construed as a potential conflict of interest.
